# An Evidential Framework for Localization of Sensors in Indoor Environments

**DOI:** 10.3390/s20010318

**Published:** 2020-01-06

**Authors:** Daniel Alshamaa, Farah Mourad-Chehade, Paul Honeine, Aly Chkeir

**Affiliations:** 1 Charles Delaunay Institute, University of Technology of Troyes, 10300 Troyes, France; farah.chehade@utt.fr (F.M.-C.); aly.chkeir@utt.fr (A.C.); 2 LITIS Lab, University of Rouen Normandie, 76130 Rouen, France; paul.honeine@univ-rouen.fr

**Keywords:** decision-making, evidence fusion, localization, WiFi RSSI

## Abstract

Indoor localization has several applications ranging from people tracking and indoor navigation, to autonomous robot navigation and asset tracking. We tackle the problem as a zoning localization where the objective is to determine the zone where the mobile sensor resides at any instant. The decision-making process in localization systems relies on data coming from multiple sensors. The data retrieved from these sensors require robust fusion approaches to be processed. One of these approaches is the belief functions theory (BFT), also called the Dempster–Shafer theory. This theory deals with uncertainty and imprecision with a theoretically attractive evidential reasoning framework. This paper investigates the usage of the BFT to define an evidence framework for estimating the most probable sensor’s zone. Real experiments demonstrate the effectiveness of this approach and its competence compared to state-of-the-art methods.

## 1. Introduction

Localization is an essential aspect in WSNs, since the knowledge of the sensor’s location is critical to process the information originating from this sensor. Many existing works have been proposed to tackle the position based localization problem. The objective is to determine the position of the sensor node according to some measured observations. A solution is to integrate a Global Positioning System or Global System for Mobile Communications (GPS-GSM) into sensor nodes. This is widely used in vehicle tracking systems [1]. However, it is not always the optimal solution because of the costs of having a GPS receiver at each node, especially when multiple objects are to be localized, as well as for the limited spatial resolution. Moreover, this technology cannot be efficiently used for indoor applications due to the large attenuation caused by buildings’ walls and ceilings. Its robustness against interference is also questionable [2].

For that reason, alternative solutions have been proposed. We consider the following approach. At first, two types of sensors are defined; anchor nodes (ANs), also called beacon nodes, of known positions, and non-anchor nodes of unknown position, to be localized. Since we consider, in the general setting, the case of moving sensors, we will refer to the non-anchor nodes as mobile nodes (MNs). The objective becomes to determine the position of any MN using collected measurements and information exchanged with the ANs. The remaining issue is to choose the appropriate enabling technology and the measurement technique. This is extremely important as it plays a vital role in the accuracy of the localization algorithm. We present hereby a brief description of the enabling technologies and the measurement techniques.

### 1.1. Enabling Technologies

Researchers have been using alternative technologies to GPS such as vision, infrared, ultrasound, ultra-wideband, Bluetooth, WiFi, etc. The vision technology is based on the processing and evaluation of video data. The video based localization can be performed either with fixed camera systems [3] or with mobile ones [4] according to the position of the camera, whether it is equipped with the ANs or the MNs, respectively. The infrared technology uses the infrared radiation to localize sensors through infrared emitters and receivers. The MN is equipped with a badge, carrying a unique identifier code (ID), that emits infrared signals at regular intervals via an infrared transmitter. Infrared receivers, placed at the ANs, detect the ID and communicate it to a localization software to determine the MN position based on the proximity between the transmitter and the receiver [5]. The ultrasound technology uses the ultrasonic waves to measure the distance between the ANs and the MN. The transmitter sends a radio signal and an ultrasonic wave at the same time. The radio signal reaches the multiple receivers almost instantaneously, providing them with the synchronization signal. The receivers then measure the time between the synchronization signal and the detection of ultrasonic waves to calculate the distance between the emitters and receivers [6]. The ultra-wideband technology is defined as a transmission from an antenna for which the emitted signal bandwidth exceeds the lesser of 500 MHz. Unlike other radio systems operating on a specific radio frequency, ultra-wideband (UWB) transmits a signal over an ultra-wide band of frequencies. The signals are transmitted for a much shorter duration with very low power spectral density, thus consuming less power than the other systems. UWB can be used in close proximity to other radio frequency signals without suffering from interference. This technology is convenient for indoor environments where the UWB signal can be easily transmitted, achieving interesting results in indoor localization applications [7]. However, a major drawback of this technology is the high cost of the UWB equipment. The Bluetooth technology is a standard for wireless personal area networks (WPANs) and operates in the 2.4 GHz band. Bluetooth has a short range and is embedded in most devices such as mobile phones, laptops, desktops, etc. For that reason, adding a new user to such network does not require any additional hardware. Bluetooth is a low cost technology, and its tags are small in size, making it an efficient technology for indoor localization. However, one of its drawbacks is that it runs the device discovery procedure at each location estimation, significantly increasing the localization latency and power consumption. This latency is unsuitable for real-time localization applications [8]. The WiFi technology uses the wireless local area network (WLAN) to estimate the location of any MN within this network. Since WLAN infrastructures are widespread in almost all indoor environments, due to the increase in demand for wireless communications, this approach is widely used for indoor localization. One of the main advantages of using WiFi over other technologies is its cost effectiveness due to the possibility to localize the position of almost every WiFi compatible device without installing any additional software. Another advantage of using WLAN is that no LoS is required. We focus here on this technology due to these advantages. Nevertheless, it was found that WiFi signal strengths are unstable and vary widely even at the same position with time, temperature, and moving objects. Another limitation of the WiFi technology is the signal attenuation of the static environment like walls, doors, and furniture [9]. For that reason, research should be carried out to solve these problems and achieve a good localization accuracy.

### 1.2. Measurement Techniques

Generally, localization techniques are split into two categories, geometrical and non-geometrical techniques. In geometric techniques, the position of the MN is estimated by compiling one or more channel characteristics, such as angle of arrival (AoA) [10], time difference of arrival (TDoA) [11], time of arrival (ToA) [12], or received signal strength indicator (RSSI) [13,14,15] into a geometric output. Equations relating the unknown position of the MN with the known positions of the ANs are derived and solved to estimate the MN position. Optimization routines such as the least squares algorithm are often used as a metric to minimize the estimation error. The RSSI based techniques exploit the attenuation of the signal strength with the traveled distance to estimate the distances separating the ANs from the MNs. Typically, ANs broadcast signals in the network, while MNs detect the broadcast signals and measure their RSSIs. The distances separating the MNs from the ANs are then estimated using the measured RSSIs and the path-loss model [16]. RSSI based techniques exhibit favorable properties with respect to power consumption, size, and cost, since no additional hardware is needed. However, distance estimation using RSSI is really challenging, since the measurements of signals’ powers can be significantly altered by the presence of additive noise, multipath fading, shadowing, and other interferences. The evidential framework was utilized for indoor localization in [17] and [18]. In [17], the authors used the belief functions theory with RSSI data; however, they relied on a pathloss model that related distance to the power of the received signal. This model has been found to suffer in terms of accuracy in practical applications especially in large areas. The model is simple, yet it does not consider the different parameters that influence the relationship between the distance and the power. This model works well in line-of-sight (LOS), while it suffers in indoor practical applications where there are rooms separated by walls and a dynamic environment where people move around continuously. In [18], the authors used the Dempster–Shafer theory for indoor localization. They implemented the AoA technique that required special hardware to measure the angle of arrival of signals and synchronization between different anchor nodes to be triangulated.

Non-geometrical techniques, which will be our adopted method in this work, do not use lines of position deduced from the estimated geometrical characteristics of the multipaths to compute the MN location. We focus here on the fingerprinting techniques as they are alternatives to the previous methods that are sensitive to the propagation conditions, such as NLoS and multipath. Indeed, fingerprinting techniques can be applied to any scenario and environment. In a preliminary step, often called offline, the area of interest is discretized into cells, and a database is built from the signal signatures. An example of such signal signatures that can be used is the RSSIs. The database can be assembled using measured data or simulated using a propagation model. In the online step, the estimated signatures at each AN are compared with the database fingerprints. This can be formulated as a regression problem [19], where the idea is to construct a model that takes the signature as an input and outputs the position of the MN [20]. The advantage of such an approach is that there is no need for a geometrical model that relates the signal strengths to traveled distances. Instead, radio-cartography is constructed by collecting measurements to cover the targeted area. It must be emphasized that the major drawbacks of fingerprinting are associated with database maintenance, sensitivity to environmental changes, and cumbersome learning. Furthermore, the offline step is often time consuming, especially if it is based on measurements [21].

Due to the ambiguity of WiFi signals and their variability in indoor environments, research is still carried out in order to enhance the performance of localization. This paper proposes a sensor localization method for indoor environments using WiFi RSSI. It is an extension of our previous works [22,23]. In [22], we studied the statistical representation of data and parametric and kernel density estimation. This allowed representing the RSSI data by a statistical model. In [23], we presented a decentralized approach for a distributed processing. This allowed reducing the complexity and thus the processing time of the localization algorithm. In this paper, we present a complete study on indoor localization using WiFi RSSI. The contributions of this work can be summarized as follows. At first, a new evidential framework for indoor localization using WiFi is proposed. In addition, a complete theoretical formulation is provided so that raw RSSI data can be utilized to determine the location of a person in real time. Moreover, a thorough experimental work is carried out to evaluate the influence of each parameter of the described theoretical framework. At last, the proposed approach is proven to outperform related state-of-the-art works in two real large scale indoor environments. In order to consider the ambiguity and uncertainty of WiFi signals, the proposed approach uses belief functions to estimate the sensors’ zones by combining evidence revealed at each AP. RSSI signals have imperfections due to reflection, diffraction, absorption, and multipath fading, which are inherent to radio waves. These phenomena are related to the environmental effects and to the presence of people and atmospheric conditions, among others. The belief functions theory allows representing and processing finely the information and its imperfections such as imprecision and uncertainty. This theory is considered as a generalization of the Bayesian probability approach where the ambiguity and uncertainty of data are taken into account. It allows assigning evidence to a set of events or hypotheses instead of a singleton one when knowledge is not sufficient to distinguish between two singleton events. A fingerprinting database is constructed that associates with each zone a set of WiFi signal strengths collected from the APs. In order to do that, we investigate both parametric and non-parametric modeling. The APs are then discounted according to their error rate. We explore both classical and contextual discounting. The discounted evidence of all APs is then combined via fusion rules, namely the conjunctive, disjunctive, and Dempster’s combination rules. In order to make decisions, the evidence assigned to subsets is transformed to singleton sets, which are the zones of the target area. We examine here both the pignistic transformation and the plausibility as criteria for decision-making. The proposed method yields a set of possible solutions, sorted in a descending order of priority, allowing for a second zone choice in case of erroneous first estimation. The performance of the proposed approach is examined in real experimental scenarios and is compared with other techniques.

## 2. An Evidential Framework for Indoor localization

Suppose an environment is divided into $N_{\;Z}$ zones denoted by $Z_{j},j\in \left\{1,2,\ldots ,N_{\;Z}\right\}$. Let $N_{\;A\;P}$ be the number of all available APs in the area of interest, denoted by $A\;P_{k},\;k\in \left\{1,2\ldots ,N_{\;A\;P}\right\}$. Let $\rho_{t}$ be the vector of size $N_{\;A\;P}$ of RSSI measurements collected by the mobile node at the instant *t* from all these APs,

(1) $$\rho_{t}=\left(\rho_{t,1},\ldots ,\rho_{t,N_{AP}}\right),$$

where $\rho_{t,k}$ is the RSSI of the signal with respect to $A\;P_{k}$ at instant *t*. Since not all APs are detected at each instant, we denote $I_{\;A\;P,t}$ the set of indices of the APs whose signals are detected by the mobile node at time *t* and $\rho_{t,k},k\in I_{\;A\;P,t}$ their measured RSSIs. The vector $\rho_{t}$ is completed with zeros at positions where the APs are not detected.

**Figure 1:**
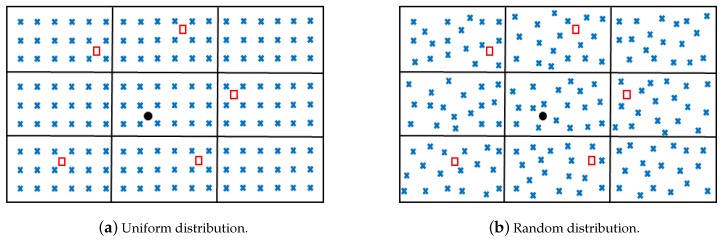
Illustration of fingerprinting configuration: × designates reference positions, □ WiFi access points, and • a mobile node.

To construct a database of WiFi fingerprints, a mobile node moves freely in each zone of the target area and measures the RSSIs of WiFi signals from all APs. Suppose $\rho_{j,k,\ell }$ corresponds to the $\ell^{\mathrm{th}}$ RSSI measured inside zone $Z_{j}$ with respect to $A\;P_{k}$. Let $N_{j}$ be the number of RSSI measurements taken in zone $Z_{j}$. This implies that for a certain zone and a given AP, a set of $N_{j}$ values is collected representing the variations of the RSSIs in this zone with respect to that AP. Let $N_{D}=\sum_{j=1}^{N_{\;Z}}N_{j}$ be the total number of measurements. A database D of $N_{D}\times N_{\;A\;P}$ RSSIs labeled to the zones is then obtained. This database describes the variability of the RSSIs within and between the zones. Figure 1 shows a grid of reference RSSI measurements collected in uniform and random distributions.

**Figure 2:**
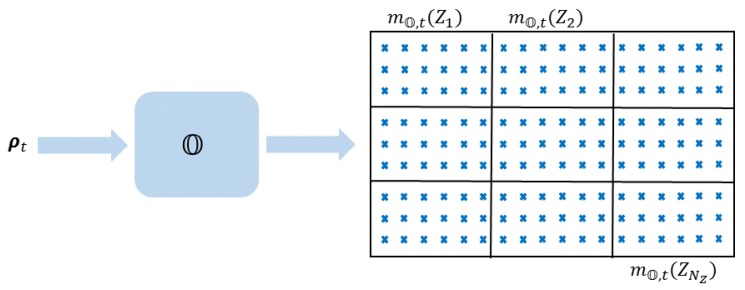
Illustration of the localization phase using the observation model O.

In the localization phase, the observation model O is used to estimate the mobile node’s zone. The mobile node to be localized measures a set of RSSIs from a certain number of APs, stores them in the vector $\rho_{t}\in R^{N_{\;A\;P}}$, and broadcasts $\rho_{t}$ in the network. The observation model O is applied to the vector $\rho_{t}$ to affiliate a confidence level instantly with each zone of the target area,

(2) $$O\left(\rho_{t}\right)=\left(m_{O,t}\left(Z_{1}\right),\ldots ,m_{O,t}\left(Z_{N_{\;Z}}\right)\right),$$

where $m_{O,t}\left(Z_{j}\right)$ is the level of confidence of having the mobile node of observation $\rho_{t}$ residing in the zone $Z_{j}$ at the instant *t*. Figure 2 illustrates the localization phase using the observation model O.

The objective of the proposed approach is to determine an observation model $O:R^{N_{\;A\;P}}\to {\left[0,1\right]}^{N_{\;Z}}$. Let $Z=\{Z_{1},\ldots ,Z_{N_{\;Z}}\}$ be the set of all possible zones, and let $P\left(Z\right)=2^{Z}$ be the set of all the subsets of Z, i.e., $P\left(Z\right)=\{\emptyset ,\left\{Z_{1}\right\},\ldots ,Z\}$. The empty set ∅ denotes the impossible zone, which means that the mobile node resides outside Z. The cardinal of P(Z) is equal to $2^{\left|Z\right|}=2^{N_{\;Z}}$, where |Z| denotes the cardinal of Z.

The observation model is constructed as follows. At first, the RSSIs collected in the database according to each AP are modeled using a distribution. We distinguish between two types of distributions, parametric and non-parametric. Supersets of single zones are also considered, and their RSSIs are also modeled. This allows us to take ambiguous information into consideration in a belief functions framework. This is important in our case especially since the measurements used are WiFi signals are unstable and ambiguous, leading to uncertain estimations and decisions. The obtained distributions are then used to set mass functions over all the subsets of P(Z). The APs, which are the sources of information, are discounted according to their error rate. Their evidence is then combined via the belief functions fusion rules, and a decision is made by associating a confidence level with each zone.

### 2.1. Statistical Representation of Data

**Figure 3:**
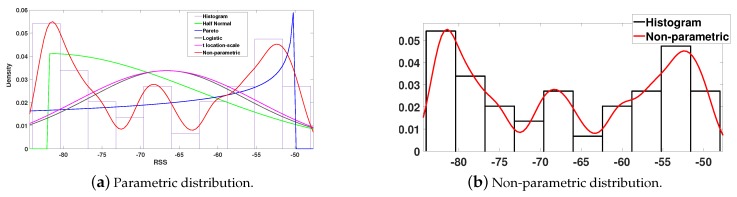
Fitting of parametric distributions in (**a**) and a KDE of Gaussian kernel in (**b**), of real data RSSIs.

Having a set of $N_{j}\times N_{\;A\;P}$ observations $\rho_{j,k,\ell },k\in \left\{1,\ldots ,N_{\;A\;P}\right\},\ell \in \left\{1,\ldots ,N_{j}\right\}$, collected in zone $Z_{j}$, the aim of this section is to fit these observations to statistical distributions that represent the variation of the RSSIs inside the zone. Although a multi-dimensional distribution can be used for this purpose, we consider here the uni-dimensional case for the following reasons. At first, uni-dimensional distributions are easier for analysis and computations, especially when the number of APs is large. In addition, they allow considering the reliability or error rate of each AP to discount the assigned evidence. Moreover, uni-dimensional distributions do not disable the process of localization when one AP, or more, is not detected for some reason. The localization can still be performed by the fusion of evidence of the detected APs only. The principle behind fitting data observations to distributions is to find the type of distribution and the values of its parameters that give the highest probability of producing the observed data. We distinguish between two types of distributions, parametric and non-parametric. The parametric modeling is realized by fitting the RSSI observations to one of the known parametric distributions. When the assumptions of the parametric distribution fail, a more general non-parametric approach is required to estimate the probability density function of the measurements [24]. The kernel density estimation (KDE) is proposed to model the RSSI measurements [25,26]. Figure 3 shows an example of real data RSSI measurements represented by their histogram, a parametric trial to fit them, and a modeling using KDE. This is an example of the failure of parametric modeling to represent the variations of RSSIs and the ability of non-parametric modeling to better represent them. A detailed description of these two types of distributions is found in [22]. Here, we only consider the final result, in either type of modeling. Both parametric and non-parametric modeling result in a distribution $Q_{A,k}\left(\cdot \right),A\in P\left(Z\right),k\in \left\{1,2\ldots ,N_{\;A\;P}\right\}$ for each set *A* with respect to each access point $A\;P_{k}$.

Mixture models in general, or Gaussian mixture models in particular, have also been shown to be very efficient in statistical representation of data, and thus can be used especially if the KDE suffers from overfitting due to the lack of data. In our case, the KDE is used for its genericity and versatility and is convenient for two reasons. The first is that the distribution of RSSIs might not follow, in many cases, any type of parametric or semi-parametric, i.e., mixture, models. This is due to the nature of RSSIs, their variability and instability, even at the same position from the same access point. This results in a random distribution, for which we assume parametric or mixture models would be restrictive in modeling it, resulting in a poor accuracy. The second reason is the facility to generate RSSI data and acquire additional observations in case the model is overfitted due to a lack of data.

### 2.2. Mass Assignment

The observation model consists of using the fitted RSSI distributions in the BFT as a framework for mass association and evidence fusion. A mass function, also called basic belief assignment (BBA), $m_{A\;P_{k},t}\left(\cdot \right)$ is a mapping from P(Z) to the interval [0,1], defined according to a certain source $A\;P_{k},k\in \left\{1,\ldots ,N_{\;A\;P}\right\}$, and satisfying:

(3) $$\sum_{A\in P(Z)}m_{A\;P_{k},t}\left(A\right)=1.$$

The mass $m_{A\;P_{k},t}\left(A\right)$ given to A∈P(Z) stands for the proportion of evidence, brought by the source $A\;P_{k}$ at instant *t*, saying that the observed variable belongs to *A* [27].

The objective is to define the APs’ BBAs, using the fitted distributions either parametrically or non-parametrically. The distribution $Q_{A,k}\left(\cdot \right)$ represents, either parametrically or non-parametrically, the variations of the RSSIs in subset *A* with respect to $A\;P_{k}$. Then, having an observation $\rho_{t,k}$ related to $A\;P_{k},k\in \left\{1,\ldots ,N_{\;A\;P}\right\}$, the mass $m_{A\;P_{k},t}\left(A\right)$ is computed as follows,

(4) $$m_{A\;P_{k},t}\left(A\right)=\frac{Q_{A,k}\left(\rho_{t,k}\right)}{\sum_{A^{\prime }\in P\left(Z\right),A^{\prime }\neq \emptyset }Q_{A^{\prime },k}\left(\rho_{t,k}\right)},\;\;A\in P\left(Z\right),A\neq \emptyset .$$

In this work, we assume that Z covers all possible zones, that is the node cannot be outside Z. This means that $m_{A\;P_{k},t}\left(\emptyset \right)=0$, for all $AP_{k}$, at any time *t*.

### 2.3. Discounting Operation

The detected APs, which are the sources of information, are not completely reliable. Indeed, each AP might yield an erroneous interpretation of evidence for some observations. This is due to the statistical modeling of the observations that is based on their occurrence in the database. Another reason is the nature of the WiFi signals, which are unstable and vary widely with various parameters. In order to correct this, one can discount the BBAs of Equation (4) by taking into account the error rate of the AP. We discuss in the following two approaches discounting the evidence assigned by the APs, classical discounting and contextual discounting.

#### 2.3.1. Classical Discounting

The reliability of a source is classically taken into account by the discounting operation, which transforms the supporting function into a weaker, less informative one [28]. The discounted BBA ${}^{\alpha }m_{A\;P_{k},t}\left(\cdot \right)$ related to $A\;P_{k}$ having an error rate $\alpha_{k}$ is deduced from the BBA $m_{A\;P_{k},t}\left(\cdot \right)$ as follows [29],

(5) $${}^{\alpha }m_{A\;P_{k},t}\left(A\right)=\left\{\begin{array}{cc} \left(1-\alpha_{k}\right)m_{A\;P_{k},t}\left(A\right), & \mathrm{if}\;\;A\in 2^{Z},\;A\neq Z;\\ \alpha_{k}+\left(1-\alpha_{k}\right)m_{A\;P_{k},t}\left(A\right), & \mathrm{if}\;\;A=Z. \end{array}\right.$$

By doing this, the amounts of evidence given to the subsets of Z is reduced, and the remaining evidence is given to the whole set Z.

To compute the error rate of a certain source $A\;P_{k}$, consider an observation $\rho_{\cdot ,k}$ being truly in *A*. The source $A\;P_{k}$ is assumed not reliable if, according to $\rho_{\cdot ,k}$, it associates more evidence with any subset other than *A*. Since the BBAs are defined using the statistical distributions related to each subset, then an AP is erroneous for all observations of *A* when $Q_{A,k}\left(\rho_{\cdot ,k}\right)$ is smaller than any $Q_{A^{\prime },k}\left(\rho_{\cdot ,k}\right)$, for any $A^{\prime }\neq A$. Let $\epsilon_{k}\left(A\right)$ be the error rate related to *A* with respect to $A\;P_{k}$. Then,

(6) $$\epsilon_{k}\left(A\right)=\int_{D_{A,k}}Q_{A,k}\left(\rho \right)d\rho ,$$

such that $D_{A,k}$ is the domain of error of subset *A* according to $A\;P_{k}$, defined as,

(7) $$D_{A,k}=\left\{\rho \;|\;Q_{A,k}\left(\rho \right)\leq \max_{A^{\prime }\in P\left(Z\right),A^{\prime }\neq A}\;\left(Q_{A^{\prime },k}\left(\rho \right)\right)\right\}.$$

The error rate $\alpha_{k}$ is then the average error of all subsets according to $A\;P_{k}$,

(8) $$\alpha_{k}=\frac{\sum_{A\in P(Z)}\epsilon_{k}\left(A\right)}{\left|P(Z)\right|}.$$

The described discounting approach requires the calculation of integrals, which might be computationally expensive. Alternatively, the error can be empirically computed, by realizing experiments and recording the number of incorrect subset estimations of each AP. The error rate $\alpha_{k}$ is then the percentage of incorrect estimations.

#### 2.3.2. Contextual Discounting

The described classical discounting approach assumes that each AP has an equal error rate with respect to all subsets. However, this is not always the case in practice, since an AP has a certain reliability regarding each subset. An AP will be more reliable to distinguish asymmetrical zones or areas, than symmetrical ones for instance. This is because in the latter case, the areas are more likely to have equal signal strengths, making them indistinguishable and thus increasing the error rate. For that reason, we consider here a contextual discounting approach to take into account the APs’ reliability [30].

Let $A=\{A_{1},\ldots ,A_{L}\}$ be a coarsening of Z, which means that $A_{1},\ldots ,A_{L}$ form a partition of Z. In this contextual model, we consider the degree of reliability of an AP conditionally on each subset $A_{l},l\in \left\{1,\ldots ,L\right\}$. For all l∈1,…,L, $\beta_{k}^{l}=1-\alpha_{k}^{l}$ represents the degree of reliability of $\;A\;P_{k}$ knowing that the observation belongs to $A_{l}$. Here, the considered partition is the set of single zones $\left\{\left\{Z_{1}\right\},\ldots ,\left\{Z_{N_{\;Z}}\right\}\right\}$, and thus, the reliability of $A\;P_{k}$ with respect to the zone, or context, $Z_{j}$ will be $\beta_{k}^{j}$. Computing the contextual discounting consists of using its expression through the disjunctive rule of combination, which is presented later in Equation (16), leading to ${}^{\alpha }m_{\;A\;P_{k}}\left(A\right)$ given by,

(9) αmAPk(A)=mAPk,t∪mAPk,t0(A),

such that $m_{\;A\;P_{k},t}^{0}\left(A\right)$ is defined as follows,

(10) mAPk,t0(A)=mAPk,t1∪mAPk,t2∪…∪mAPk,tNZ(A),

where each $m_{\;A\;P_{k},t}^{j},j\in \left\{1,\ldots ,N_{\;Z}\right\}$, is defined as,

(11) $$m_{\;A\;P_{k},t}^{j}=\left\{\begin{array}{cc} (1-\alpha_{k}^{j}), & \mathrm{if}\;\;A=\emptyset ;\\ \alpha_{k}^{j}, & \mathrm{if}\;\;A=A_{l};\\ 0, & \mathrm{otherwise}. \end{array}\right.$$

The error rate $\alpha_{k}^{j}\left(A\right)$ of subset *A* such that the truth is $Z_{j}$ with respect to $A\;P_{k}$ is computed as,

(12) $$\alpha_{k}^{j}\left(A\right)=\int_{D_{A,k}}Q_{A,k}\left(\rho \right)d\rho ,$$

such that $D_{A,k}$ is the domain of error of subset *A* according to $A\;P_{k}$, defined as,

(13) $$D_{A,k}=\left\{\rho \;|\;Q_{\{Z_{j}\},k}\left(\rho \right)\leq \max_{A^{\prime }\in P\left(Z\right),A^{\prime }\neq A}\;\left(Q_{A^{\prime },k}\left(\rho \right)\right)\right\}.$$

However, computing these multi-dimensional integrals might be computationally expensive. For that reason, the reliability rate $\beta_{k}^{j}=1-\alpha_{k}^{j}$, of $\;A\;P_{k}$, is obtained by finding the percentage of correct subset determination such that the truth is $Z_{j}$. To this end, we construct a confusion matrix that describes the performance of the AP on a set of $N^{l}$ measurements to be tested in each zone. A confusion matrix $C=c_{mn},m\in \left\{1,\ldots ,N_{\;Z}\right\}$ and $n\in \{1,\ldots ,N_{\;Z}\}$, is a table where each line *m* corresponds to a decision in favor of $Z_{m}$ and each column *n* corresponds to the case where the truth is $Z_{n}$. The general term $c_{mn}$ is equal to the number of tested measurements of $Z_{n}$ that have been assigned to $Z_{m}$ by $\;A\;P_{k}$. The reliability rate is the percentage of correct estimations, computed as $\beta_{k}^{m}=\frac{c_{mn}}{N^{n}}$. The error rate is thus $\alpha_{k}^{m}=1-\frac{c_{mn}}{N^{n}}$.

### 2.4. Fusion of Evidence

The mass functions ${}^{\alpha }m_{A\;P_{k},t}\left(\cdot \right)$ are defined according to the RSSI vector $\rho_{t,k},k\in I_{\;A\;P,t}$ retrieved from a certain number of APs. It is important to combine these mass functions in a meaningful way. This allows the fusion of the different pieces of evidence in order to result in a final decision. Combination rules are considered to be a major building block of the BFT. These rules aim to merge all these mass functions successively in order to obtain a mass function representing all available evidence [31]. A survey of different combination rules was given in [32].

#### 2.4.1. Dempster’s Rule

Dempster’s rule of combination was first introduced by Dempster [33] and then reinterpreted by Shafer [28] as a basis for the BFT. The objective of this rule is to normalize belief functions that are defined over the same frame of discernment and are based on independent arguments or bodies of evidence. The mass function obtained through Dempster’s combination rule ⨁ is as follows,

(14) $$m_{⨁,t}\left(A\right)=\frac{\sum_{\begin{array}{c} A^{\left(k\right)}\in P\left(Z\right)\\ \cap_{k}A^{\left(k\right)}=A \end{array}}\prod_{k\in I_{\;A\;P,t}}{\;}^{\alpha }m_{A\;P_{k},t}\left(A^{\left(k\right)}\right)}{1-\sum_{\begin{array}{c} A^{\left(k\right)}\in P\left(Z\right)\\ \cap_{k}A^{\left(k\right)}=\emptyset \end{array}}\prod_{k\in I_{\;A\;P,t}}{\;}^{\alpha }m_{A\;P_{k},t}\left(A^{\left(k\right)}\right)},$$

for all the subsets A∈P(Z), where $A^{\left(k\right)}$ is the subset *A* with respect to the source $A\;P_{k}$. This fusion rule leads to a more informative and specialized mass function [28].

#### 2.4.2. Conjunctive Rule

The conjunctive rule of combination is an adaptation of Dempster’s rule where unnormalized belief functions are allowed. The conjunctive rule can be also applied by considering only the numerator of Equation (14), avoiding the normalization factor,

(15) m∩,t(A)=∑A(k)∈P(Z)∩kA(k)=A∏k∈IAP,tαmAPk,t(A(k))

#### 2.4.3. Disjunctive Rule

The disjunctive rule of combination is applied when only one of several pieces of evidence holds. If the APs are conflicting, the previous rules generate counter-intuitive results. For that reason, the disjunctive rule is considered to combine the obtained evidence. By using the disjunctive rule, it is enough that at least one AP is reliable to acquire logical results. Therefore, the aggregated mass attributed to each subset *A* is computed as,

(16) m∪,t(A)=∑A(k)∈P(Z)∪kA(k)=A∏k∈IAP,tαmAPk,t(A(k)).

Since the union $\cup_{k}A^{\left(k\right)}$ is never empty unless all the subsets $A^{\left(k\right)}$ are empty, there is no conflict resulting from the disjunctive rule of combination, and therefore, there is no need for normalization.

### 2.5. Confidence Based Zone Estimation

Decision-making under uncertainty is an important problem in real-world applications. The BFT aims to model a decision maker’s subjective evaluation of evidence [34]. It allows one to express partial beliefs when complete information is not available. Some methods for using the BFT in decision making have been studied in [35,36,37]. In order to make decisions based on the BFT, Smets [38] argued that beliefs first need to be transformed to probabilities. Some solutions exist to ensure the decision-making within the theory of belief functions. The best known is the pignistic probability proposed by the transferable belief model (TBM). The computed mass function or BBA consists of an interpretation of the information brought by the observations at a given time *t*. It is a kind of belief or evidence, which is not a probability measure. An adequate notion of the BFT to attribute a confidence level to singleton sets is the pignistic level [39]. Smets [40] argued that in order to make decisions, the belief represented by the BBA and held at the credal level must induce a probability function at the pignistic level. This is known as the pignistic transformation. It is defined as follows [41],

(17) $$BetP_{t}\left(A\right)=\sum_{A\subseteq A^{\prime }}\frac{m_{⨁,t}\left(A^{\prime }\right)}{|A^{\prime }|},$$

where *A* is a singleton of P(Z). The mass obtained by Dempster’s rule is shown in Equation (17), but the conjunctive and disjunctive rules can be equivalently used. The pignistic level is equivalent to the probability of having the observation belonging to the considered subset. Using this concept, the level of confidence associated with each zone by the basic observation model at each instant *t* can be computed as follows,

(18) $$m_{O,t}\left(Z_{j}\right)=BetP_{t}\left(\left\{Z_{j}\right\}\right),j\in \left\{1,\ldots ,N_{\;Z}\right\}.$$

Other criteria for decision-making exist like the maximum of credibility and the maximum of plausibility [38]. The plausibility is an important notion in the belief functions theory. It can be computed from the mass function as follows,

(19) $$pl_{t}\left(A\right)=\sum_{A^{\prime }\cap A\neq \emptyset }m_{t}\left(A^{\prime }\right).$$

The plausibility is a measure of the maximum support of evidence that could be given to *A* [42]. The level of confidence associated with each zone is computed as follows,

(20) $$m_{O,t}\left(Z_{j}\right)=pl_{t}\left(\left\{Z_{j}\right\}\right),j\in \left\{1,\ldots ,N_{\;Z}\right\}.$$

For any decision-making criteria, the observation model O is deduced,

(21) $$O\left(\rho_{t}\right)=\left(m_{O,t}\left(Z_{1}\right),\ldots ,m_{O,t}\left(Z_{N_{\;Z}}\right)\right),$$

$m_{O,t}\left(\cdot \right)$ being the level of confidence assigned either using the pignistic transformation or the plausibility. The zone having the highest confidence level is thus selected. We also obtain a sorted list of zones, allowing for a second choice in the case of first erroneous zone estimation.

## 3. Experimental Results

Real experiments were conducted in order to evaluate the performance of the proposed method. In the following, the experimental setups are first introduced. Afterwards, the performance of the method is assessed. The influence of modeling, discounting, combination, reference positions, and number of zones and APs is studied. Finally, the performance of the proposed method is compared with other techniques.

### 3.1. Experimental Setups

**Figure 4:**
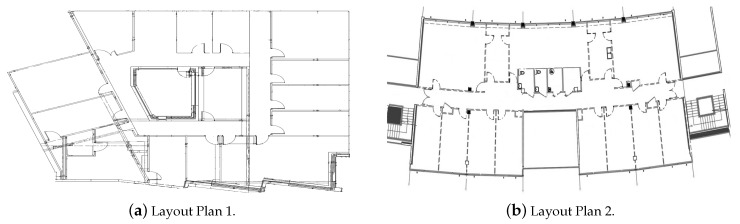
The Living Lab in (**a**) and the first floor of the statistical and operational research department in (**b**) at the University of Technology of Troyes, France.

The real experiments were realized in a WLAN environment at the first floor of the Statistical and Operational Research Department and the Living Lab at the University of Technology of Troyes, France, as shown in Figure 4. The layouts had an approximate area of 500 and 550 m${}^{2}$ and were partitioned into 19 and 21 zones, respectively. A personal computer, with a “WiFi scanner” software, distinguished the APs of the network throughout their MAC addresses. It measured then the RSSIs of their transmitted signals. We used 23 and 38 AP networks in the first and second target area, respectively. Here, we distinguished an AP network from a physical AP. On the same physical AP, several networks existed (different frequencies, different target users, etc). In each experiment, a set of 50 measurements was taken in each zone, of which 30 were randomly used to construct the databases, and the others were kept for test and validation. The user, carrying a personal computer for data acquisition, moved around while regularly collecting measurements in random positions and orientations of the personal computer. Data were also acquired in a normal dynamic environment while all people were moving around and practicing their life in a regular manner. We assumed that these conditions would be similar to when the method was applied in the real environment. Computations were conducted on Version 7.11.2(R2010B) of MATLAB on a laptop with Microsoft Windows 7 and Intel Core i7 CPU. Table 1 summarizes the experimental setup parameters.

The dynamic environment is one of the major challenges for the robustness of fingerprinting approaches. To deal with this issue, the fingerprinting database was acquired in the normal dynamic environment, which was expected to be in the daily activities. An important application of this work is the localization of elderly people in indoor environments such as homes, nursing homes, clinical care centers, rehabilitation suites, psychiatric clinics, etc. In such environments, it is expected that the real dynamic environment would be similar, to a certain extent, to that when the database was collected. To study the performance of the proposed approach in various conditions, we collected test observations in Layout Plan 1 after one month and after five months of the date of original data acquisition. We thus refer to Experiment 1 as the results obtained on the observations collected on the same day as the training database. We refer to Experiments 2 and 3 as applying the method to test observations after 1 month and after 5 months, respectively, of the data for the database acquisition, using the same original database for training. We refer to Experiment 4 as the experiment in the second target area, Layout Plan 2.

#### 3.1.1. Influence of Discounting and Combination

Two important concepts for zoning based localization in a belief functions framework are the discounting and the combination of APs’ evidence. In this section, we study the influence of the discounting technique, classical or contextual, and the combination rule, Dempster’s, conjunctive, or disjunctive, on the performance of the proposed approach. Table 2 indicates the overall accuracy of the proposed method, obtained by the various combinations of discounting techniques and combination rules. As the table shows, the contextual discounting carried an enhancement of 3 to 4% as compared with the classical discounting. This was due to considering the reliability of the APs per area or zone, which is important in the case of localization using RSSIs. On the other hand, a slight advantage of the disjunctive rule was recorded when evaluating the combination rules in Layout Plan 1. This is because the used APs were found to be conflicting. This was not the case of Layout Plan 2, where the conjunctive rule of combination outperformed other ones. We concluded that both Dempster’s and conjunctive rules resulted in a better accuracy. However, in the presence of conflicting evidence between APs, they resulted in counter-intuitive results, and thus in a relatively lower accuracy. In such cases, the disjunctive rule of combination was found to be better.

#### 3.1.2. Influence of Modeling and Reference Positions

Another important issue is the modeling of the RSSI measurements. The parametric modeling might not be always appropriate to represent the variations of the observations in a certain zone with respect to some AP. For that reason, we explore the influence of a non-parametric modeling or a KDE on the performance of the proposed approach in this section. Table 3 provides a comparison of the performance of the proposed approach between a parametric and a KDE modeling. As the table shows, the KDE had an important influence on the overall accuracy. This was less significant in a uniform distribution of reference positions setting, where the variations of the RSSI measurements were found to follow parametric distributions at a better significance level than in the case of a random distribution setting. As a result, it is important to try first a parametric modeling and verify if the obtained results are satisfactory. If not, a KDE can be adopted as previously mentioned.

We now consider a random distribution of the reference RSSI positions instead of a uniform grid. Table 3 shows the overall accuracy of the proposed approach in the case of a random distribution of reference positions, considering both parametric and non-parametric modeling. Compared with the results obtained when a uniform distribution of reference positions was considered, one can see that the overall accuracy decreased with the use of random distributions. This was due to the fact that a uniform grid allowed a better coverage of the region of interest, while a random distribution did not always guarantee a good coverage of the region. Nevertheless, the results were still acceptable and satisfactory, and random distributions could still be used for localization when uniform grids could not be achieved.

#### 3.1.3. Influence of the Number of Zones and Decision-Making Criteria

**Figure 5:**
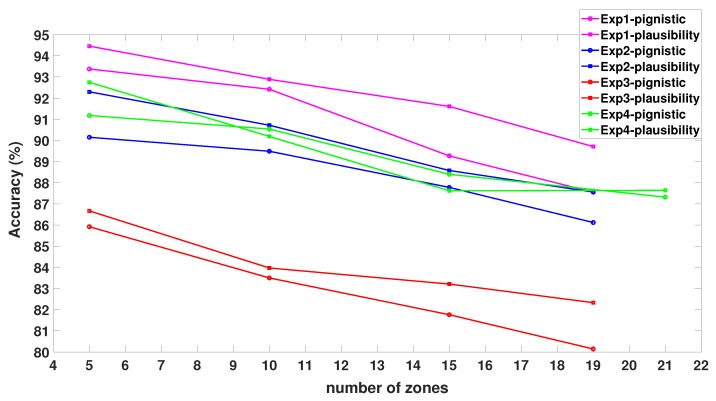
Influence of the number of zones and decision-making on the overall accuracy (%).

To study the influence of the number of zones $N_{\;Z}$ on the performance of the proposed approach, we varied the number of zones from 5 to 19 in the first three experiments corresponding to Layout Plan 1 and from 5 to 23 in the fourth experiment corresponding to Layout Plan 2. Figure 5 shows the performance of the proposed approach as a function of the number of zones. As the table shows, the localization accuracy decreased as the number of zones increased. This was due to the inability of the proposed observation model to assign discriminating evidence to the widely overlapping mass functions representing the different zones. We also explored the influence of the decision-making criteria. Figure 5 compares the performance of the proposed approach for a decision made based on either the pignistic transformation or the plausibility. The figure shows a superior performance when the decision was made based on maximum plausibility rather than pignistic transformation.

### 3.2. Comparison with Other Techniques

In this section, we compare the proposed method to other well known techniques. For example, a connectivity based localization algorithm was proposed in [43]. Such algorithms do not rely on collected measurements, and they are thus range free. In this case, the sensor’s location is given as the intersection of the ranges of the APs detected by the sensor. The problem that we tackled here can be also formulated as a multi-class classification technique where conventional classification techniques such k nearest neighbors (KNN), neural networks (NN), and support vector machines (SVM) could be applied. In Table 4, we present the overall accuracy and the localization processing time of the proposed approach as compared to the various described techniques. For neural networks, we considered an input layer with 23 neurons (38 for Experiment 4) corresponding to the number of APs. We considered an output layer with 19 neurons (21 for Experiment 4) corresponding to the number of zones. We considered one single hidden layer with 21 neurons (30 for Experiment 4), as an average between the number of neurons of input and output layers. The radial basis function was used as an activation function. For SVM, we considered a Gaussian kernel. The hyper-parameters were tuned using grid search. For KNN, the number of neighbors used was found to be 13 (15 for Experiment 4). All parameters were fine tuned using ten fold cross-validation. As the table shows, the proposed method outperformed, in the most cases, other techniques in terms of overall accuracy. The advantage of the connectivity method was that it was independent of the collected measurements, so it was not influenced by the dynamic environment. We noticed that the proposed method was influenced by the dynamic environment. After one and five months, the overall accuracy decreased from 90% to 87% and 82%, respectively. This required an update of the original training database.

We also studied the dependency of the localization method on the number of APs. In fact, a localization method that requires a large number of APs to achieve a high localization accuracy is not preferred, since it is practically unfeasible in most of the cases due to the unavailability of sufficient APs in the network, or due to the installation cost. In order to study the influence of this parameter on the performance of the described approaches, Table 5 shows the overall accuracy as a function of the number of available APs. We can note that the connectivity method for example was highly sensitive to the density of APs, as it required a higher number of APs to achieve a good accuracy. The proposed method was less sensitive to this parameter and still achieved a good performance at this level, with a decrease of only 7% in overall accuracy, upon a decrease in the number of detected APs from 23 to five.

## 4. Conclusions

We proposed a zoning based localization approach in a belief functions framework using WiFi fingerprints. Different types of modeling were considered, namely the parametric and non-parametric distributions, to describe the variations of the RSSI observations statistically. The obtained distributions were used to define mass functions over the zones with respect to the available APs in the network. Once a new observation was carried for localization, the constructed mass functions were used to assign a mass for each zone with respect to each AP. The evidence attributed by each AP was then discounted according to its error rate. Pieces of evidence were combined afterwards using fusion rules, and a confidence level was assigned to each zone through the maximum of plausibility and pignistic transformation. The zone having the highest confidence was supposed to be the zone where the mobile node resided. Experimental results showed that the KDE was better when the observations failed to fit a parametric distribution. In addition, an advantage was noted for the disjunctive rule of combination when APs yielded conflicting evidence. The conjunctive rule of combination resulted in a better performance when the AP were not conflicting. The contextual discounting showed an enhancement in the overall accuracy as compared to the classical discounting. This was due to the fact that the reliability of APs varied with respect to the zone. An enhancement was carried out by the maximum plausibility decision-making criteria as compared to the pignistic transformation. However, the proposed approach was found to be vulnerable to the number of zones in the target area. The overall accuracy decreased as the number of zones increased. Another disadvantage of the proposed approach was its vulnerability to the dynamic environment. After one and five months, the accuracy of the proposed approach decreased significantly. The proposed approach achieved a good overall accuracy, outperforming other techniques. As future work, we aim to consider a hierarchical clustering technique in order to reduce the number of zones being assigned evidence at any moment. We will also investigate the combination of mobility models for the proposed approach in order to enhance the overall accuracy. The belief functions theory is an interesting framework to combine evidence from different natures of sources of information. In addition, we aim to find a solution for the dynamic environment, either through a manual update of the database or through transfer learning. 

## Figures and Tables

**Table 1:** Experimental setup parameters.

Parameter	Notation	Value	
		Layout 1	Layout 2
Number of zones	$N_{\;Z}$	19	21
Number of APs	$N_{\;A\;P}$	23	38
Number of measurements per zone	$N_{j}$	30	30

**Table 2:** Influence of the discounting techniques and the combination rules on the overall accuracy (%).

Accuracy (%)	Discounting							
	Classical				Contextual			
Combination rule	1	2	3	4	1	2	3	4
Dempster’s	83.75	83.48	79.22	82.94	86.89	86.20	81.41	85.68
Conjunctive	83.85	83.07	80.45	83.89	87.27	86.88	82.56	87.64
Disjunctive	85.50	85.19	79.62	80.80	89.71	87.56	82.33	83.57

**Table 3:** Influence of the type of modeling and distribution of reference positions on the overall accuracy (%).

Accuracy (%)	Type of Modeling							
	Parametric				KDE			
Positions	1	2	3	4	1	2	3	4
Uniform	86.54	84.08	80.94	84.95	89.71	87.65	82.33	87.64
Random	84.50	82.64	77.13	80.45	87.28	86.51	80.28	84.88

**Table 4:** Comparison between methods in different experiments in terms of accuracy (%).

Technique	Number of Experiment			
	1	2	3	4
Connectivity	84.17	84.05	84.42	82.56
KNN	81.88	78.24	74.19	82.31
NN	84.72	84.51	81.73	84.98
SVM	85.55	83.48	82.82	84.76
Proposed	89.71	87.65	82.33	87.64

**Table 5:** Comparison between different methods in terms of overall accuracy (%), as a function of the number of APs.

Technique	Number of Detected APs			
	5	10	15	23
Connectivity	65.56	69.44	76.67	84.17
KNN	70.22	74.78	77.17	81.88
NN	77.78	80.00	81.39	84.72
SVM	78.61	80.83	82.78	85.55
Proposed	82.22	83.33	85.27	89.71
